# Gut microbiota mediated the individualized efficacy of Temozolomide via immunomodulation in glioma

**DOI:** 10.1186/s12967-023-04042-5

**Published:** 2023-03-16

**Authors:** Xiaoying Hou, Hongzhi Du, Yufei Deng, Haiping Wang, Jinmi Liu, Jialu Qiao, Wei Liu, Xiji Shu, Binlian Sun, Yuchen Liu

**Affiliations:** 1 Wuhan Institute of Biomedical Sciences, School of Medicine, Jianghan University, Wuhan, China; 2 Cancer Institute, School of Medicine, Jianghan University, Wuhan, China; 3 School of Pharmacy, Hubei University of Chinese Medicine, Wuhan, China

**Keywords:** Temozolomide, Fecal microbiome, Functional Metabolomics, Individualized efficacy, Glioma

## Abstract

**Background:**

Temozolomide (TMZ) is the preferred chemotherapy strategy for glioma therapy. As a second-generation alkylating agent, TMZ provides superior oral bio-availability. However, limited response rate (less than 50%) and high incidence of drug resistance seriously restricts TMZ’s application, there still lack of strategies to increase the chemotherapy sensitivity.

**Methods:**

Luci-GL261 glioma orthotopic xenograft model combined bioluminescence imaging was utilized to evaluate the anti-tumor effect of TMZ and differentiate TMZ sensitive (S)/non-sensitive (NS) individuals. Integrated microbiomics and metabolomics analysis was applied to disentangle the involvement of gut bacteria in TMZ sensitivity. Spearman’s correlation analysis was applied to test the association between fecal bacteria levels and pharmacodynamics indices. Antibiotics treatment combined TMZ treatment was used to confirm the involvement of gut microbiota in TMZ response. Flow cytometry analysis, ELISA and histopathology were used to explore the potential role of immunoregulation in gut microbiota mediated TMZ response.

**Results:**

Firstly, gut bacteria composition was significantly altered during glioma development and TMZ treatment. Meanwhile, in vivo anti-cancer evaluation suggested a remarkable difference in chemotherapy efficacy after TMZ administration. Moreover, 16s rRNA gene sequencing and non-targeted metabolomics analysis revealed distinct different gut microbiota and immune infiltrating state between TMZ sensitive and non-sensitive mice, while abundance of differential gut bacteria and related metabolites was significantly correlated with TMZ pharmacodynamics indices. Further verification suggested that gut microbiota deletion by antibiotics treatment could accelerate glioma development, attenuate TMZ efficacy and inhibit immune cells (macrophage and CD8α^+^ T cell) recruitment.

**Conclusions:**

The current study confirmed the involvement of gut microbiota in glioma development and individualized TMZ efficacy via immunomodulation, hence gut bacteria may serve as a predictive biomarker as well as a therapeutic target for clinical TMZ application.

**Supplementary Information:**

The online version contains supplementary material available at 10.1186/s12967-023-04042-5.

## Background

According to the latest epidemiological statistics, central nervous system cancer will be one of the top ten death causes of cancer in 2022 [1]. Among the brain and other nervous system tumors, malignant glioma (accounts for more than 80%) has been a devastating type due to its extreme malignancy, high mortality rate and recurrence risk, which lead to social and family burden [2]. Temozolomide (TMZ) is a second-generation oral alkylating agent for clinical malignant glioma therapy. It acts primarily through DNA methylation and interferes with DNA replication, thereby induces cell cycle arrest at G2/M and results in cancer cell death [3]. Based on the latest version of Guidelines for the diagnosis and treatment of glioma (2022), TMZ is always the standard strategy for malignant glioma [4].

Compared with radiotherapy, TMZ treatment for glioma can increase patients’ median survival period, progression-free period as well as the patients’ life quality. However, only less than 50% of the glioma patients positively respond to TMZ therapy, drug resistance is the main issue for failure of TMZ therapy [5]. Except for the classical resistance mechanism induced by over-expression of O^6^-methylguanine-DNA methyltransferase (MGMT), recent studies also indicated that factors such as autophagy, isocitrate dehydrogenase (IDH), miRNAs, TP53/p53, EGFR signal pathway are involved in TMZ efficiency [6–8]. Nevertheless, there still lacks of effective strategy to overcome individualized TMZ effect and enhance the positive response in glioma patients.

Increasing evidences suggested that gut microbiota and derived metabolites could modulate cancer development and chemotherapy efficacy [9]. Researchers proposed that specific gut bacteria could affect drug efficacy directly through drug metabolism or indirectly through immune system regulation [10]. Inherent heterogeneity of human gut microbiota is an established factor for individualized drug efficacy. And individuals harbor radically different collections of microbiome may result in vastly different responses to the same treatment [11]. Our previous studies also confirmed that gut microbiota *Prevotalla* and related metabolite 3-Oxocholic acid attenuated chemotherapy sensitivity of FOLFOX in colon cancer. Meanwhile, *Akkermansia muciniphila* transplantation combined with FOLFOX significantly enhanced its chemotherapy efficacy [12, 13]. *Yoshua* reported that the gut microbiota could be altered during glioma development and TMZ treatment [14], yet the underlying mechanism and whether the individualized gut microbiota plays a role has to be revealed. Therefore, it is essential to further investigate the mechanism by which gut flora contribute to individualized efficacy of TMZ in glioma.

This study intends to reveal the potential role of gut microbiota in glioma development and individualized efficacy of TMZ using integrated microbiomics and metabolomics analysis. A glioma orthotopic xenograft mice model was constructed to distinguish TMZ Sensitive (S) individuals from Non-Sensitive (NS) individuals. Meanwhile, 16s rRNA sequencing, non-targeted metabolomics as well as Spearman correlation analysis were combined to explore the involvement of gut microbiota in glioma development, TMZ treatment and individualized TMZ response between S and NS mice. Subsequently, ELISA and histopathology were applied to explore the potential role of immune regulation in gut microbiota mediated chemotherapy response of TMZ. Finally, broad-spectrum antibiotic combined treatment was performed to confirm the role of gut microbiota and immunomodulation in the individualized anti-glioma effect of TMZ. In brief, our study provided potential predictive biomarker and targets for the individualized therapy of TMZ.

## Material and methods

### Chemicals and reagents

TMZ (T127425) was purchased from Aladdin (Shanghai, China). Ampicillin (MB1507), Metronidazole (MB2200), Neomycin sulfate (MB1716) and Vancomycin (MB1260) were purchased from Meilunbio (Dalian, China).

### Cell culture

The mice glioma cell line GL261 was obtained from the American Type Culture Collection (Rockville, USA), the cells were cultured in DMEM (Gibco, Grand Island, USA) with 10% Fetal Bovine Serum (Gibco). Luci-GL261 was generated through transduction of lentiviral vectors encoding firefly luciferase (LV16-NC) purchased from GenePharma Co., Ltd. (Shanghai, China) supplied with 5 μg/mL polybrene (Sigma), and screened by and puromycin (5 μg/mL, Sigma-Aldrich). All the cells were incubated at 37 °C in a humidified atmosphere with 5% CO_2_.

### Intracranial tumor implantation and TMZ treatment

Five to six-week-old male C57BL/6 mice (18–22 g) were provided by the Beijing Vital River Laboratory Animal Technology Co. Ltd. (Beijing, China) with the permission number SCXK (Jing) 2021–0006. The study was conducted in accordance with the standards established by the Medical Ethics Committee of Jianghan University. All the mice were housed in temperature-controlled environment (24 ± 2 °C) under a 12/12 h-dark/light cycle.

Glioma orthotopic xenograft model was constructed based on previously reports [14, 15]. Briefly, mice were anesthetized by isoflurane inhalation, 4 μL Luci-GL261 cell suspension (about 10^5^ cells) was intracranial injected at striatum (1-mm anteroposterior and 1.5-mm lateral to the bregma, 3.5-mm below the cortical surface) (day 7). Tumor formation was confirmed 7 days after injection (day 14) via the in vivo spectral real-time imaging system (IVIS, USA), then the mice were randomly divided into groups (10 mice/group), TMZ (50 mg/kg) was daily gavaged to TMZ group [16], tumor development was monitored by bioluminescence imaging.

### 16s rRNA gene sequencing analysis

Bacterial DNA extraction and quantification was performed as we described previously [12]. After checked by 1% agarose gels electrophoresis for integrity, PCR amplification was performed spanning the V4 hypervariable regions (515F and 806R) of the bacteria 16s rRNA gene and sequenced on NovaSeq6000 (Illumina, San Diego, USA). Raw Tags were filtered by Qiime (http://qiime.org/scripts/split_libraries_fastq). High-quality sequences were clustered into Operational Taxonomic Units (OTUs) with similarity ≥ 97% by USEARCH UPARSE. Then, OTUs were classified into kingdom, phylum, class, order, family and genus levels, and eventually an OTU table was created. α and β diversity was performed by Qiime (Version 1.9.1). MetaStat analysis was applied to identify the differentially abundant taxa between the groups (it was considered statistically significant when *p* < 0.05). Phylogenetic Investigation of Communities by Reconstruction of Unobserved States (PICRUSt) was employed to predict the functional profiling of microbial communities based on the Kyoto Encyclopedia of Genes and Genomes (KEGG) pathway database.

### Metabolomics analysis

Fecal samples from mice were detected by non-targeted metabolomics analysis. Methanol was added to the fecal homogenate to precipitate protein and extract metabolites. After two times centrifugation (4 °C, 12000 rpm, 10 min), the supernatant was collected and analyzed by liquid chromatography tandem mass spectrometry on a Triple TOF-6600 mass spectrometer. Compound separation was performed on a Waters Acquity UPLC HSS T3 C18 column (2.1 *100 mm, 1.8 µm). The mobile phase was consisting of (A) 0.1% formic acid in water and (B) 0.1% formic acid in acetonitrile. The eluting gradient was described as follows: mobile phase A was decreased from 95 to 10%, within 11 min, maintained at 10% A for 1 min, then brought back to 95% and maintained there for 2 min. Flow rate was 0.4 mL/min. Column temperature was set at 40 °C; Injection volume was 2 μL; Electrospray ionization (ESI) source in both positive and negative modes. Interface voltage was 5.5 kV for positive mode and − 4.5 kV for negative mode. Metabolites were annotated by comparing the m/z values, formulae and the MS/MS fragmentations with to online databases, such as HMDB (http://www.hmdb.ca), the Mass Bank (http://www.massbank.jp) and METLIN Metabolite (http://metlin.scripps.edu), etc. Further confirmation was done by comparing with available standard compounds with respect to retention time, accurate mass as well as mass spectra.

Quality control (QC) was generated by pooling equal aliquot of each sample and was processed together with actual samples. QC was injected every ten samples in the analytical sequence to check the robustness of the non-target metabolomics workflow. Data nomarlization was carried out by area normalization. The variable importance in projection (VIP) generated from orthogonal partial least-squares-discriminant analysis (OPLS-DA) models and *p* values from non-parametric Mann–Whitney U test (SPSS 20.0) were used to determine whether a feature is significantly different between the two groups or not. Only features with VIP > 1 and *p* < 0.05 were considered for metabolite annotation.

### Identification of TMZ sensitive (S) and non-sensitive (NS) individuals

TMZ Sensitive (S) and Non-Sensitive (NS) individuals were defined by tumor inhibition rate: (1-RTF_T_/RTF_M_) × 100%, while RTF_T_ represents the Relative Total Flux (Flux at day 21/ Flux at day 14) of TMZ group, RTF_M_ represents the Relative Total Flux (Flux at day 21/ Flux at day 14) of Model group. TMZ treated mice with inhibition rate > 30% were recognized as TMZ Sensitive (S group), and inhibition rate < − 200% were recognized as TMZ Non-Sensitive (NS group).

### Histopathology

Tumor tissues were formalin fixed and paraffin embedded. Sections were then subjected for hematoxylin and eosin (HE) staining and immunohistochemistry (Ki67, F4/80 and CD8α) as we previously reported [17, 18].

### Enzyme-linked immunosorbent assay (ELISA)

The secreted level of IL-1β and Tumor Necrosis Factor-α (TNF-α) in mice serum was measured using ELISA kits (Bioswamp, Wuhan, China) according to the manufacturer’s instructions.

### Antibiotics treatment

Tumor-bearing mice were treated with broad-spectrum antibiotics cocktail (ABX, 0.5 g/L Vancomycin, 1 g/L Neomycin sulfate, 1 g/L Metronidazole and 1 g/L Ampicillin) into their sterile drinking water to deplete gut microbiota as previously reported [19].

### Statistical analysis

Spearman’s correlation analysis was applied to test the correlation between fecal bacteria levels and fecal metabolite intensities (SPSS 20.0). Data analysis and graphing were performed by GraphPad Prism 8 software (GraphPad Software Inc., La Jolla, CA, USA). The results were presented as mean ± SD, independent unpaired two-tailed Student’s t test was performed to evaluate the differences between two groups, unless otherwise specified.

## Results

### Multi-omics study indicated that glioma development leads to gut microbiota dysbiosis in mice

**Fig. 1:**
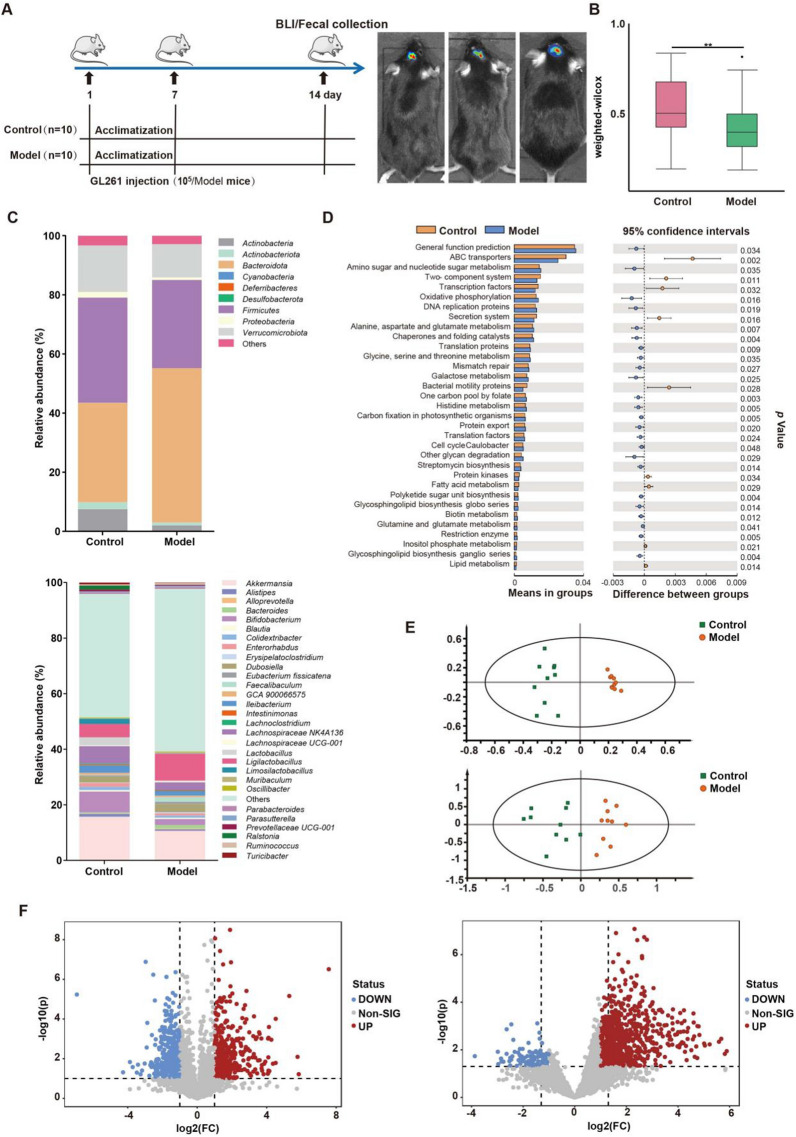
Glioma induces gut microbiota and related metabolites dysbiosis of mice. **A** Workflow of Glioma orthotopic xenograft model construction and representative bioluminescence images (BLI) of mice in model group at day 14. **B** The β-diversity indexes evaluated by weighted-wilcox of gut microbiota between Control and Model groups. **C** Taxonomic distributions of bacteria from Control and Model groups at phylum and genus level. **D** Differently abundant of KEGG pathways of the gut microbiota in Control and Model mice. **E** OPLS-DA score plot based on LC–MS ( +) (R^2^X = 0.242, R^2^Y = 0.967, Q^2^ = 0.69) and LC–MS (-) (R2X = 0.491, R2Y = 0.801, Q2 = 0.629) data. **F** Volcano plots of differential metabolites with fold change > 2 and *p* < 0.05 based on LC–MS ( +) and LC–MS (-) between Control and Model groups

To explore the involvement of gut microbiota in glioma development, we constructed a Luci-GL261 glioma orthotopic xenograft model. The xenograft model was verified by bioluminescence imaging at day 14 (Fig. 1A and Additional file 1: Fig. S1). At the same time, fecal samples from Control and Model group were collected for 16s rRNA sequencing analysis (day 14).

The α-diversity indexes showed that glioma development did not lead to significant change in the overall microbial community richness (Chao1, Shannon and Simpson) (Additional file 1: Fig. S2). However, the β-diversity between Control and Model mice was prominently different, suggesting that the dysbiosis of gut microbiota structure and composition is induced by glioma development (Fig. 1B). Specifically, as shown in Fig. 1C, *Firmicutes* (accounting for an average of 35.58%) was the dominated phylum in the Control group, while *Bacteroides* (accounting for an average of 52.16%) was the most prominent in Model mice. At genus level, similarity percentage analysis revealed that *Akkermansia*, *Bifidobacterium*, *Ligilactobacillus*, etc. were key factors contributing to the variability between Control and Model mice (Additional file 1: Fig. S3). Furthermore, KEGG pathway enrichment analysis showed enriched level of ABC transporters in Control mice, while Model group had increased metabolic pathways such as polyketide sugar unit biosynthesis, Glycosphingolipid biosynthesis and amino acid metabolism (*p* < 0.01) (Fig. 1D).

Recent studies have highlighted the essential role of gut bacterial metabolites in the interaction of gut microbiota and the host [20, 21]. Therefore, the potential function of microbiota related metabolites in glioma development was investigated by comparing fecal samples from Control and Model group using non-targeted metabolomics analysis. A tight clustering of the QCs in the PCA score plots derived from LC–MS ( +) and LC–MS (-) datasets was observed, indicating that the metabolomics methods were robust (Additional file 1: Fig S4A). OPLS-DA model was then established to explore the differences between the two groups (Fig. 1E). Permutation test with 500 iterations demonstrated that the OPLS-DA models were not overfitting (Additional file 1: Fig S4B). Only the features satisfied with VIP > 1, *p* < 0.05 and fold change > 2 were selected for metabolite annotation (Fig. 1F). A total of 64 metabolites were identified, mainly including amino acids, short-chain fatty acids (SCFAs), fatty acids, bile acids, dipeptides, etc. (Additional file 1: Table S1). In addition, KEGG enrichment analysis highlighted different levels of tryptophan/ glutamate metabolism, linolenic acid metabolism, fatty acid biosynthesis pathways between Control and Model group (Additional file 1: Fig. S4C). Taken together, multi-omics study indicated that gut microbiota is greatly variable during glioma development.

### TMZ treatment altered intestinal flora distribution and increased its diversity

**Fig. 2:**
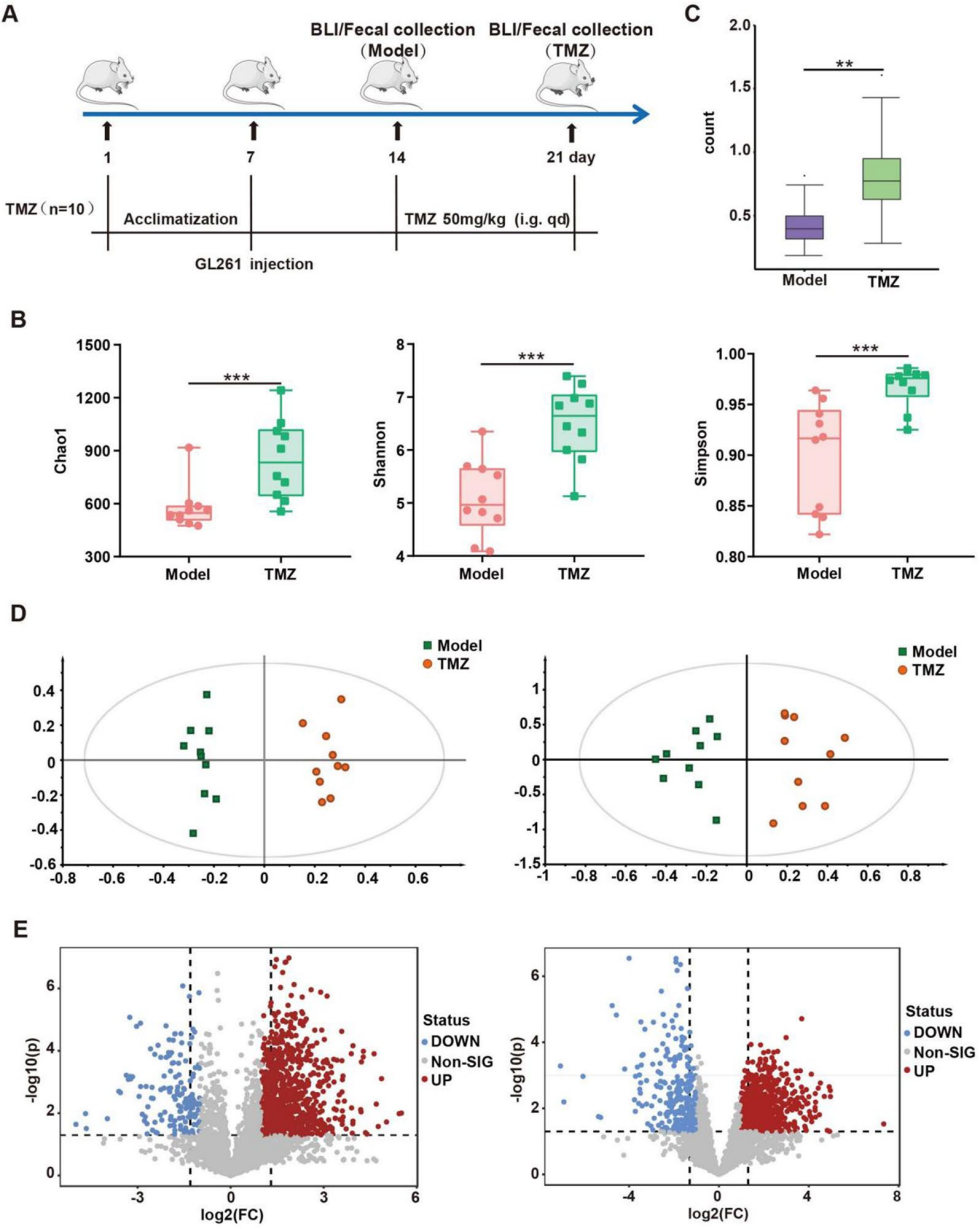
TMZ administration leads to significantly changed intestinal flora distribution **A**Workflow of glioma orthotopic xenograft model construction and TMZ treatment. **B** The α-diversity indexes of Chao1, Shannon and Simpson of gut microbiota in mice before (Model) and after TMZ (TMZ) treatment. **(C)** The β-diversity indexes evaluated by weighted-wilcox of gut microbiota between Model and TMZ treated mice. **D** OPLS-DA score plot based on LC–MS ( +) (R^2^X = 0.262, R^2^Y = 0.972, Q^2^ = 0.798) and LC–MS (-) (R^2^X = 0.642, R^2^Y = 0.867, Q^2^ = 0.708) data. **E** Volcano plots of differential metabolites with fold change > 2 and *p* < 0.05 based on LC–MS ( +) and LC–MS (-) between Model and TMZ treated mice

In light of the observed association between gut microbiota dysbiosis and glioma, the effects of TMZ treatment, a glioma standard chemotherapy on mice gut flora were next explored. Fecal samples before (Model, collected at day 14) and after (TMZ, collected at day 21) TMZ therapy were collected for 16s rRNA sequencing and metabolomics analysis, respectively (Fig. 2A). Consistent with reported results [22, 23], TMZ therapy reversed the dysbiosis of gut microbiota induced by glioma. After TMZ treatment, the α-diversity indexes of Chao1, Shannon and Simpson were significantly increased (*p* < 0.001) in mice, suggesting an elevated microbial community richness and diversity (Fig. 2B). Meanwhile, the β-diversity was also significantly different between the two groups (*p* < 0.01) (Fig. 2C). At phylum level, the TMZ mice was dominated by *Firmicutes* (accounting for an average of 52.04%), whereas *Bacteroides* was the dominant bacteria in the Model group (Additional file 1: Fig. S5A). Specifically, the abundance of 17 bacterial genera including *Akkermansia*, *Ruminococcus*, *Lactobacillus*, etc. was significantly different between Model and TMZ mice (Additional file 1: Fig. S5B, C). Furthermore, KEGG pathway enrichment analysis showed different activities of amino acid, lipid, glucose and nucleotide metabolism between the two clusters (Additional file 1: Fig. S6).

Non-targeted metabolomics analysis of fecal samples further supported the altered gut bacterial metabolic profiling after TMZ therapy (Fig. 2D). QC samples were clustered very well in PCA score plots (Additional file 1: Fig. S7A), permutation test (500 times) demonstrated that the models were not overfitting (Additional file 1: Fig. S7B). Between Model and TMZ mice, 61 metabolites involved in amino acid, oligopeptide, cholic acid, short/long-chain fatty acid metabolism were finally annotated as significantly different (VIP > 1, *p* < 0.05 and fold change > 2) (Additional file 1: Table S2). These data confirmed that TMZ reversed the gut microbiota dysbiosis induced by glioma orthotopic xenograft and increased gut microbiota diversity.

### Significantly different gut microbiota structure and metabolites between pre-dose fecal samples from TMZ sensitive and non-sensitive individuals

**Fig. 3:**
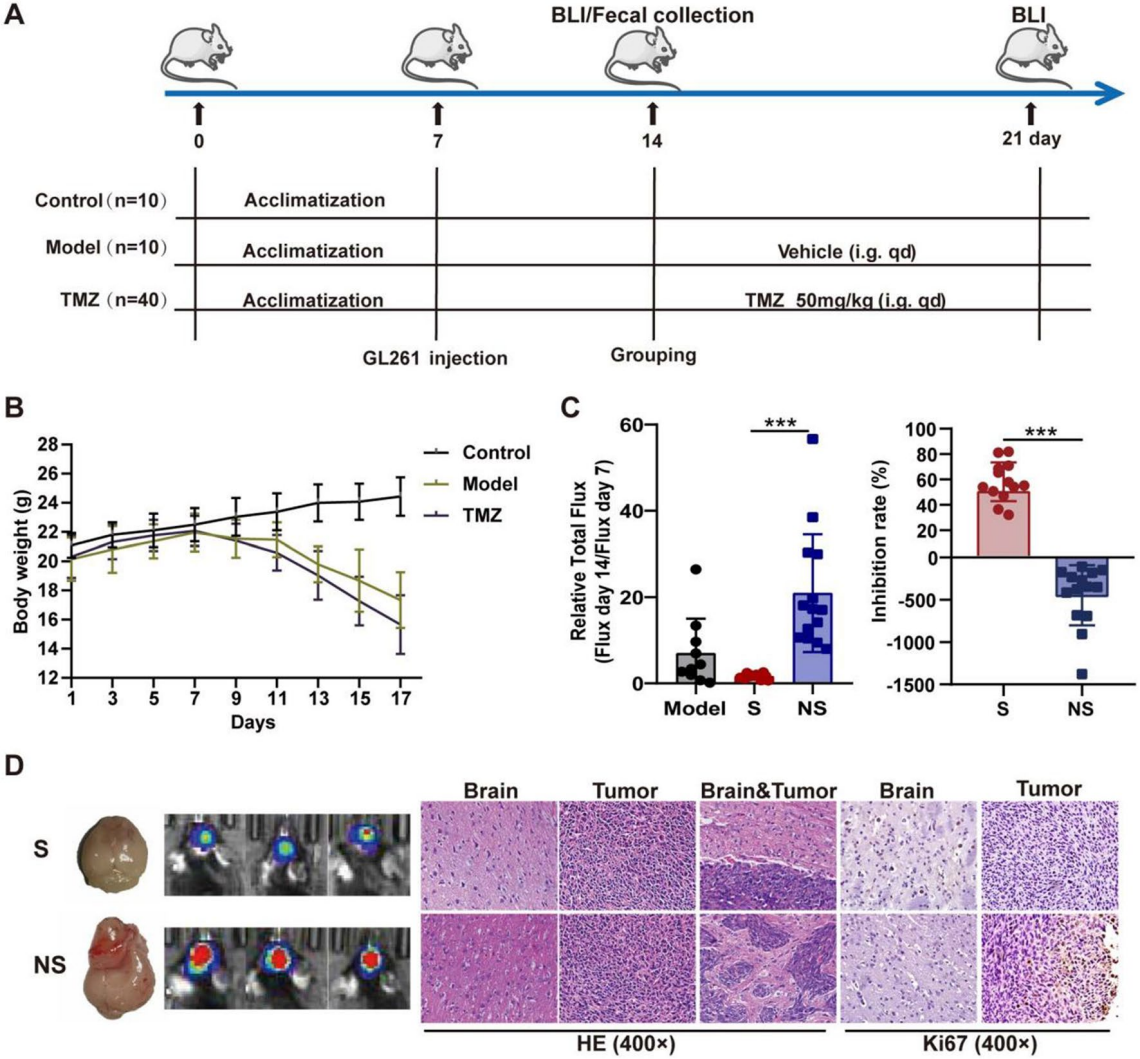
Identification of TMZ Sensitive (S) and Non-Sensitive (NS) individuals. **A** Workflow of Glioma orthotopic xenograft model construction and TMZ treatment. **B** Effect of glioma development and TMZ administration on mice body weight. **C** TMZ efficacy evaluated by Relative Total Flux (Flux day 14/Flux day 7) and tumor inhibition rate on TMZ S (n = 13) and NS (n = 14) mice, respectively. **(D)** Representative photos, BLI images, HE staining and immunohistochemistry (Ki67) of S and NS mice

**Fig. 4:**
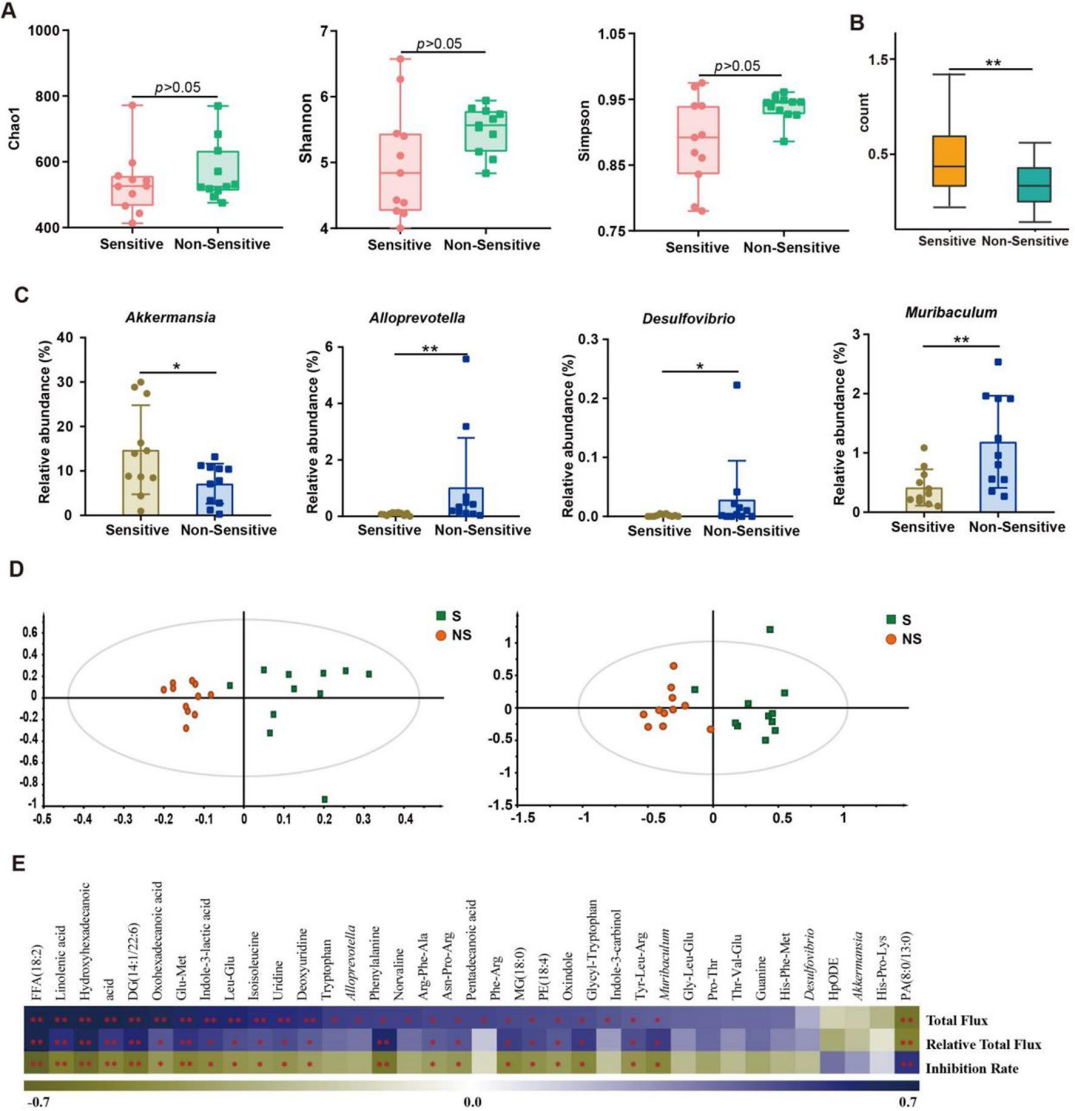
Different gut microbiota distribution and metabolites may contribute to individualized TMZ efficacy. **A** The α-diversity indexes of Chao1, Shannon and Simpson of TMZ S and NS mice gut microbiota. **B** The β-diversity indexes evaluated by weighted-wilcox of gut microbiota between TMZ S and NS individuals. **C** Significantly changed bacterial genera between S and NS mice. **D** OPLS-DA score plot based on LC–MS ( +) (R^2^X = 0.236, R^2^Y = 0.794, Q^2^ = -0.522) and LC–MS (-) (R^2^X = 0.475, R^2^Y = 0.808, Q^2^ = 0.569) data. **E** Heatmap of Spearman correlation coefficient between pharmacodynamics indices after TMZ treatment and abundance of changed bacterial genera/metabolites. The intensity of the colors represents the degree of association between the level of pharmacodynamics indices and abundance of changed bacterial genera/metabolites determined by Spearman’s correlations. The *p*-values < 0.05 were considered statistically significant, *p < 0.05, **p < 0.01

As the first-line strategy in glioma chemotherapy, high heterogeneity of TMZ response is responsible for poor prognosis in some patients [24]. Recent studies revealed that multiple factors including MGMT mutation, tumor immune microenvironment, inflammasome and PI3K/Akt pathways could contribute to individualized TMZ efficacy [25–27], whereas there still lack of strategies to increase the chemotherapy sensitivity. Our results have sufficiently confirmed the involvement of gut microbiota in glioma development and TMZ therapy. However, whether gut microbiota could mediate the sensitivity of tumor-bearing mice to TMZ remains unknown. Therefore, Luci-GL261 glioma orthotopic xenograft model was subsequently constructed (day 7), mice were randomly divided into Model group (n = 10) and TMZ group (n = 40) 7 days after tumor injection (day 14) (Fig. 3A). Both tumor development and TMZ treatment led to significant decreased body weight of tumor-bearing mice (Fig. 3B). Based on the tumor inhibition rate (evaluated by relative total flux = Flux at day 21/ Flux at day 14), TMZ treated mice was divided into TMZ sensitive (S group with inhibition rate > 30%, n = 13) and TMZ Non-Sensitive (NS group with inhibition rate < -200%, n = 14). As show in Fig. 3C, TMZ treatment showed remarkable difference in the anti-tumor effect between the S group and NS group. Meanwhile, cancer metastasis and Ki67 levels were also prominently inhibited in TMZ S mice (Fig. 3D). These results confirmed that TMZ could be efficient in some individuals inoculated with traditional TMZ resistant GL261 cells, indicating other key factors were involved in TMZ efficacy. We subsequently explored whether gut microbiota and related metabolites contribute to the sensitivity of TMZ treatment in glioma by 16s rRNA gene sequencing analysis and non-targeted metabolomics analysis on pre-dose fecal samples from S and NS group. We found no significant difference of α-diversity (Chao1, Shannon and Simpson) between S and NS groups (Fig. 4A), whereas the gut microbiota distribution was significantly different when analyzed with β-diversity index (Fig. 4B). Further analysis revealed that the abundance of *Bacteroides* in NS individuals (59.05%) was remarkably higher than that in S group (38.97%) (Additional file 1: Fig. S8A). This result is consistent with the higher abundance of *Bacteroides* in Model group (52.16%) than that in Control group (33.54%) (Fig. 1C). Further screen showed that the abundance *Akkermansia*, *Alloprevotella*, *Muribaculum*, *Desulfovibrio* was the most different among gut microbiota taxa between S and NS mice (Fig. 4C and Additional file 1: Fig. S8B). Meanwhile, metabolomics analysis revealed significantly different metabolic levels related to gut microbiota (Additional file 1: Fig. S9A, B and Fig. 4D). KEGG pathway based function prediction also suggested markedly different levels of tryptophan metabolism, steroid biosynthesis and sphingolipid metabolism between pre-dose fecal samples from S and NS group (Additional file 1: Fig. S9C). Spearman correlation analysis suggested the a significant association between the abundance of changed bacteria and metabolites in fecal samples (Additional file 1: Fig. S10). Subsequent analysis revealed that the abundance of differential gut microbiota and metabolites was significantly correlated with pharmacodynamic evaluation indexes (Fig. 4E and Additional file 1: Fig. S11). In short, different distribution and function of gut microbiota from TMZ sensitive and non-sensitive individuals was characterized in this section, whereas the potential role of gut bacteria in TMZ efficacy and underlined mechanism requires further exploration.

### Mice with different response to TMZ therapy exhibit significant variation of immune infiltration

**Fig. 5:**
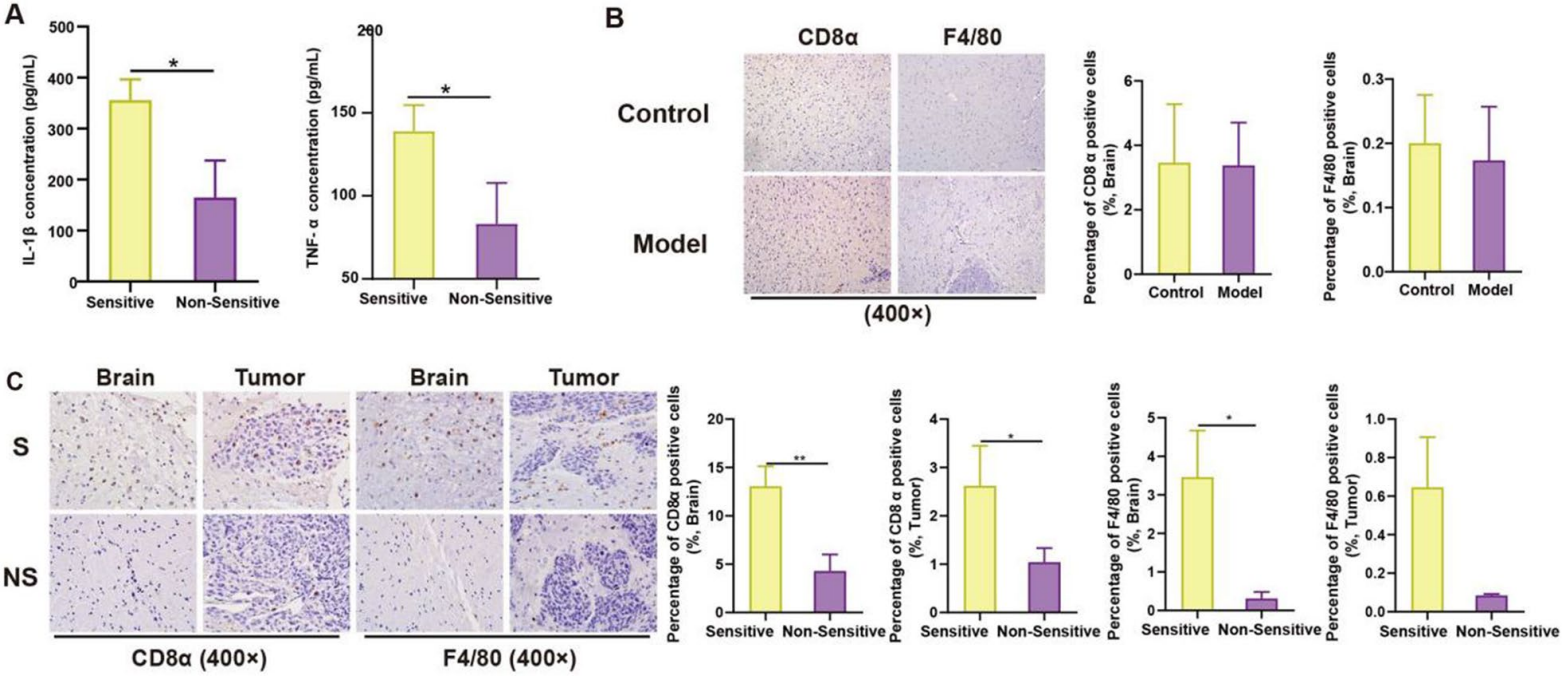
Different immune infiltration of TMZ sensitive and non-sensitive mice. **A** Levels of IL-1β and TNF-α in S and NS mice serum. Immunohistochemistry and quantification of CD8α, F4/80 in mice from Control, Model group **(B)**, and from S and NS group **(C)**. The *p*-values < 0.05 were considered statistically significant, *p < 0.05

Gut microbiota could mediate disease development and drug response through immune system regulation [28]. And it is known that immune escape is one of the characteristics in glioma [29]. We speculate that gut bacteria influence TMZ sensitivity via immune modulation. Therefore, we studied serum concentrations of IL-1β and TNF-α from TMZ sensitive and non-sensitive mice collected at day 21, respectively. As shown in Fig. 5A, levels of IL-1β and TNF-α were significantly increased in sensitive individuals, indicating the immunosuppression induced by glioma was reversed in sensitive mice. To further confirm the speculation, infiltration degrees of macrophage (F4/80) and cytotoxic T lymphocytes (CD8α) at day 21 were assessed by immunohistochemistry. While it was not significantly changed between Control and Model mice (Fig. 5B), the percentage of macrophage and cytotoxic T lymphocytes was obviously higher in TMZ sensitive mice in both brain&tumor tissues (Fig. 5C). Taken together, our study suggested an increased immune infiltration in TMZ sensitive individuals.

### ABX administration restricted TMZ efficacy and recruitment of immune cells

**Fig. 6:**
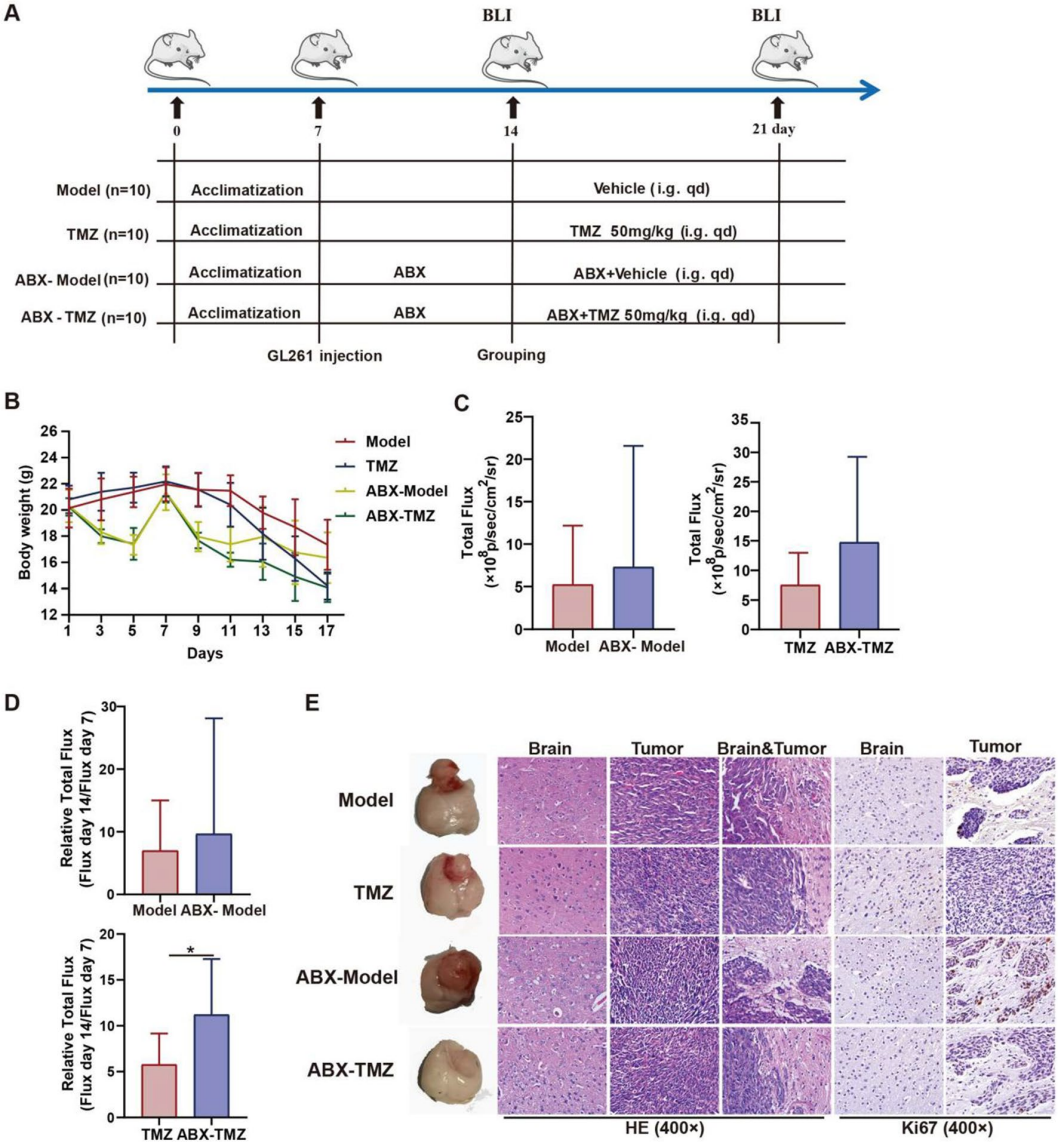
Effects of broad-spectrum antibiotic (ABX) treatment on TMZ efficacy. **A** Workflow of the ABX treatment experiment. **B** Effect of TMZ and ABX administration on mice body weight. **C** Total flux of mice in different groups. **D** Chemotherapy efficacy evaluated by Relative Total Flux (Flux day 14/Flux day 7). **E** Representative photos, HE staining and immunohistochemistry (Ki67) of mice in different groups

**Fig. 7:**
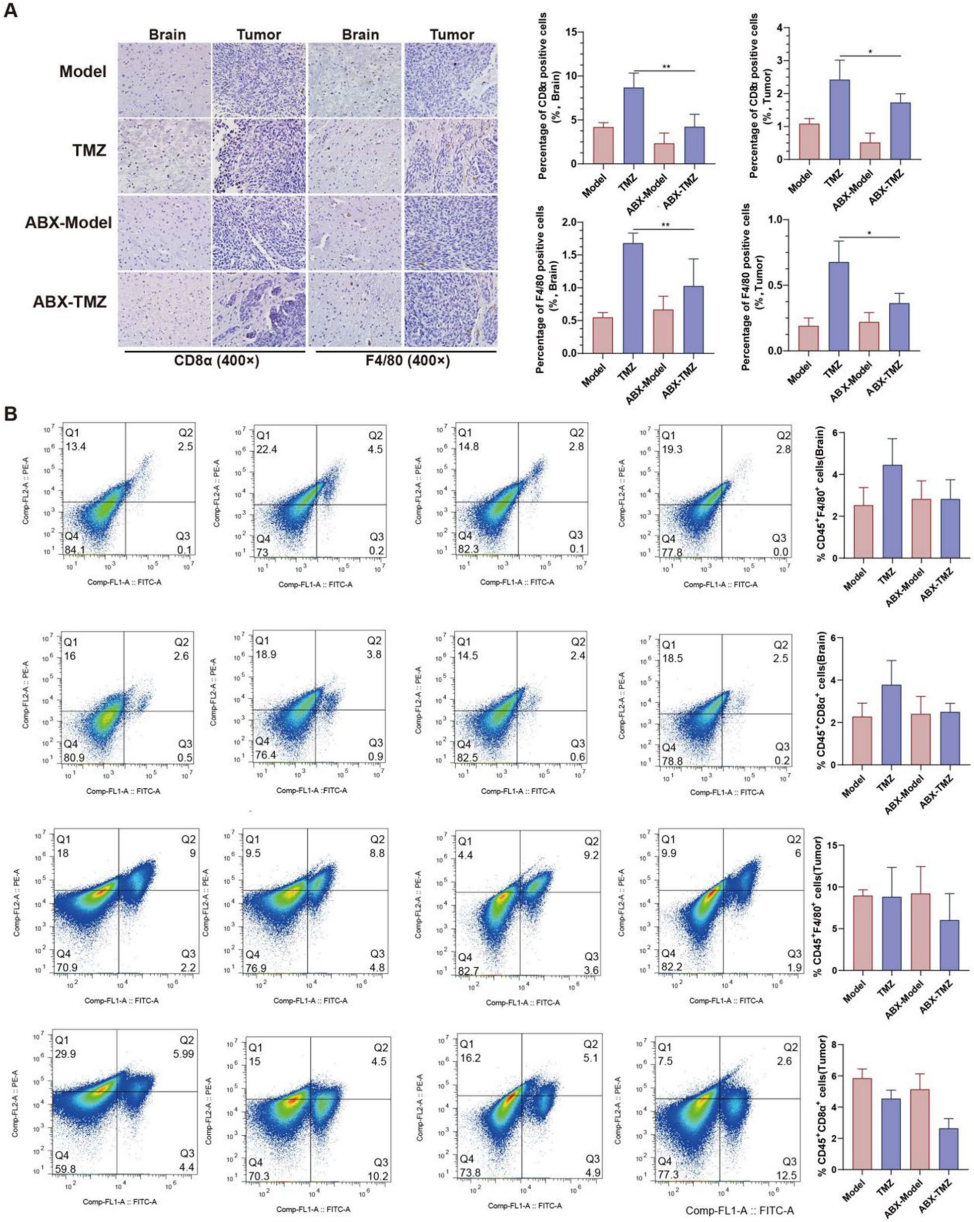
Effects of broad-spectrum antibiotic (ABX) treatment on immune infiltration of tumor-bearing mice. Immunohistochemistry **(A)** and Flow Cytometry Analysis **(B)** of F4/80 and CD8α in mice

After confirming the difference of gut microbiota structure and immune infiltration between TMZ sensitive and non-sensitive mice, we wanted to further investigate the involvement of gut microbiota in individualized TMZ sensitivity. Broad-spectrum antibiotics cocktail (ABX), a classical combination of antibiotics consisted of ampicillin, metronidazole, neomycin sulfate, and vancomycin. And ABX has been widely accepted to be sufficient to deplete all detectable commensal bacteria. Thus, ABX treatment was applied to eradicate gut microbiota of tumor-bearing mice (Fig. 6A), and the ABX treatment led to a further decrease of body weight in mice as expected (Fig. 6B). The subsequent evaluation of anti-tumor effect of TMZ with/without gut flora showed that ABX slightly promoted the glioma development (*p* > 0.05), while significantly attenuated TMZ efficacy (*p* < 0.05) (Fig. 6C, D). Meanwhile, the invasive capability and Ki67 levels of tumor were obviously elevated after gut microbiota deletion (Fig. 6E). These results confirmed the involvement of gut bacteria in glioma development and the TMZ anti-glioma efficacy. Furthermore, IHC indicated a decreased recruitment of macrophage and cytotoxic T lymphocytes in brain & tumor tissues after ABX combination (TMZ *versus* ABX-TMZ) (Fig. 7A). Results from Flow Cytometry Analysis suggested an increased recruitment of macrophage and cytotoxic T lymphocytes in brain after TMZ treatment (Model *versus* TMZ), while a decreased level of macrophage and cytotoxic T lymphocytes after ABX combined TMZ treatment in tumor (ABX-Model *versus* ABX-TMZ), indicating that gut microbiota does mediate TMZ efficacy through immune regulation (Fig. 7B). To sum up, microbiomics integrated with metabolomics proved gut microbiota could influence the individualized TMZ efficacy via immunomodulation in glioma orthotopic xenograft model.

## Discussion

**Fig. 8:**
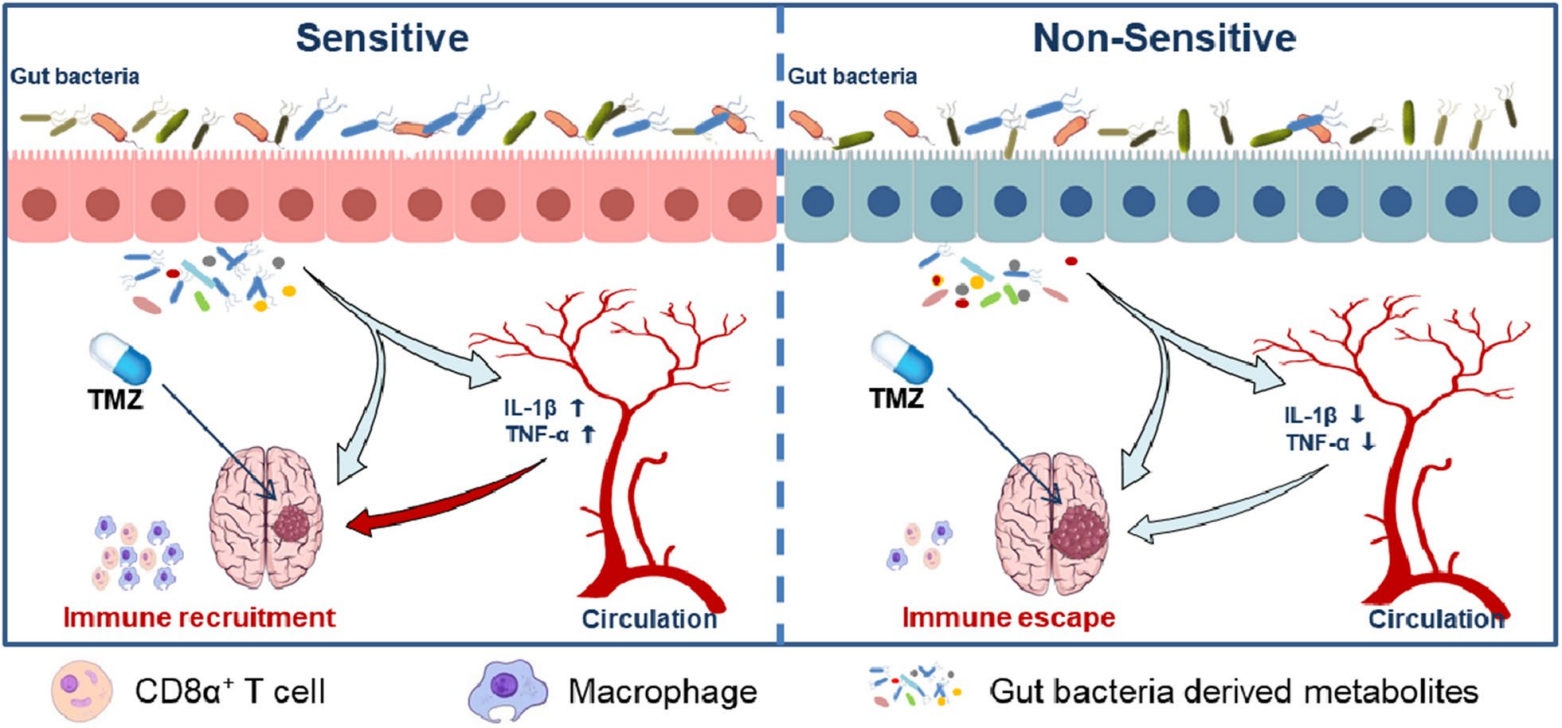
Schematic diagram illustrating the gut microbiota mediate individualized TMZ efficacy through immune regulation

Temozolomide, as the first-line chemotherapy for glioma, has shown superiority in the treatment of glioma than radiotherapy alone. However, the relative low response rate of TMZ restricts its therapeutic efficiency as well as the prognosis of patients. It is important to reveal the underlying key factors involved in the TMZ chemotherapy efficacy. In the current study, a significant alteration of gut microbiota was confirmed during glioma development and TMZ treatment. Meanwhile, glioma orthotopic xenograft mice model displayed distinct sensitivity to TMZ therapy. And we found distinguishable variation of gut bacterial distribution and immune infiltration levels from the pre-dose fecal samples between TMZ sensitive and non-sensitive mice. Furthermore, ABX treatment further confirmed the involvement of gut microbiota mediated immunomodulation in the individualized TMZ efficacy. Taken together, our study suggests gut microbiota influence individualized TMZ efficacy through immune regulation (Fig. 8).

Consistent with the previous study [23], we found both glioma development and TMZ treatment affect the gut microbiota distribution and related metabolites of mice (Figs. 1, 2), indicating that gut microbiota play an essential role in glioma pathological progress and TMZ therapy response. It is intriguing to understand underlying mechanism of gut microbiota in TMZ efficacy. Our study found that TMZ is still effective in up to 30% of the mice harboring TMZ resistant glioma (GL261 cells) (Fig. 3), suggesting the involvement of other key factors in TMZ efficacy. Therefore, we analyzed gut microbe distribution and metabolism levels between sensitive and non-sensitive mice. We found a significant different structure of gut flora between the two groups. And the abundance of differential gut bacteria and related metabolites was significantly correlated with pharmacodynamic evaluation of TMZ (Fig. 4). Further experiment confirmed the critical role of gut microbiota in individualized efficacy of TMZ by showing that ABX pre-treatment could significantly attenuate the anti-tumor effect of TMZ. A growing body of evidence increasingly validates the presence of heterogeneous gut microbiota and its effect in chemotherapy [30, 31]. Our findings support the phenomena in glioma development and individualized sensitivity to TMZ treatment.

It is known that gut microbiota can influence chemotherapy indirectly through immune regulation [20]. Inflammatory pathways such as NF-κB pathway, STAT3 signaling, etc. and related inflammatory factors (ILs, TNFs, IFNs, etc.) are both important for gut bacteria mediated host immune response [20, 32]. Especially it is known that IL-1β and TNF-α are important mediators for the interactions between gut-brain axis [33]. In this study, different serum levels of inflammatory factors (IL-1β, TNF-α) was observed between TMZ S and NS individuals. The presence of immune cells such as macrophage and CD8^ +^ T cell differs too between brain tissue and tumor (Fig. 5). These results confirm that gut microbiota influence TMZ treatment efficacy through immunomodulation. Further investigation revealed that ABX treatment decrease the immune infiltration in glioma tumor-bearing mice thus reverse the anti-tumor effect of TMZ (Fig. 7). The data firmly prove the involvement of immune system in gut bacteria mediated individualized TMZ response.

Gut microbiota derived metabolites reflect gut microbiome, and play pivotal roles in the interactions between gut microbe and the host [34]. In our study, KEGG pathway based function prediction suggests significantly different levels of tryptophan metabolism between S and NS group (Additional file 1: Fig. S9). Previous studies have confirmed gut microbiota could convert dietary derived tryptophan into tryptamine, kynurenine and indole derivatives. Tryptophan and related metabolites are established key microenvironmental factors in shaping the immune microenvironment to affect glioma development [35, 36]. Specifically, gut microbiota could transform tryptophan into kynurenine via IDO1. Both kynurenine and IDO1 are confirmed as important regulators for body immune homeostasis [37].And AHR, an important sensor for bacterial indoles and kynurenine, also plays pivotal roles bacterial mediated tumor immune microenvironment [38]. Meanwhile, *Yang* has confirmed that tryptophan metabolic enzyme tryptophan hydro xylase 1 could promote glioma progression through serotonin/L1CAM/NF-κB signaling pathway [39]. Based on the significant correlation between tryptophan metabolism and glioma development as well as TMZ efficacy (Additional file 1: Fig. S9, S4C and Fig. 4E), we speculate that IDO1 and AHR are the potential targets in bacterial tryptophan metabolism. Our preliminary results showed that the level of AHR was significantly higher in Control mice compared to Model mice, which is also increased in TMZ Sensitive mice when compared with Non-sensitive individuals (Additional file 1: Fig. S12). These results indicate that the altered tryptophan metabolites may be involved in glioma development and TMZ efficacy. However, the biological function of gut microbe derived tryptophan and related metabolites in TMZ efficacy as well as the underlying mechanism need further investigation.

The current study highlights the essential role of gut microbiota and related immunomodulation in the individualized response to TMZ therapy. Thus, the role of gut microbiota on TMZ efficacy requires further verification based on clinical samples and patient derived xenograft models. Meanwhile, 4 bacterial genera were identified as significantly different between Sensitive and Non-Sensitive mice for the first time, it is interesting to explore the effect of the 4 differential bacterial taxa transplantation on the anti-tumor effect of TMZ. In addition, this study only confirmed the involvement of immunomodulation in gut microbiota mediated TMZ efficacy, though the underlying mechanism requires more investigation.

In conclusion, our study identifies the critical role of gut microbiota in the anti-cancer effect of TMZ. In particular, different gut microbiota distribution and immune infiltration were identified between TMZ sensitive and non-sensitive individuals. Furthermore, ABX treatment accelerated glioma development, reversed the anti-tumor effect of TMZ and aggravated the immunosuppression induced by cancer. This study revealed the involvement of gut microbiota in individualized TMZ efficacy through immunomodulation, and hence gut microbiota may serve as a predictive biomarker or a therapeutic target for glioma treatment. Elucidating the respective roles of gut microbiota and their metabolites may offer opportunities for precision medicine intervention using temozolomide in glioma therapy.

## Supplementary Information

**Additional file 1: Figure S1.** 3D images for glioma orthotopic xenograft mice. **Figure S2.** The α-diversity indexes of Chao1 (A), Shannon (B) and Simpson (C) of gut microbiota between Control and Model groups. **Figure S3.** Similarity percentage (SIMPER) analysis based on Bray-Curtis was used to study bacterial species (Top 10) contributing to the variability between Control (C) and Model (M) groups. **Figure S4.** Non-target metabolomics analysis of fecal samples from Control and Model mice. **(A)** QC samples were clustered very well in PCA score plots derived from LC-MS (+) and LC-MS (-) datasets. (B) Permutation test result (500 times) of OPLS-DA models constructed from LC-MS (+) and LC-MS (-) data. (C) Differently enrichment of KEGG pathways in Control and Model mice. **Figure S5.** Taxonomic distributions of bacteria before (Model) and after TMZ (TMZ) treatment at phylum level (A) and genus level (B); (C) Significantly changed bacterial genera between Model and TMZ mice evaluated by MetaStat analysis. **Figure S6.** Differently abundant of KEGG pathways of the gut microbiota before (Model) and after (TMZ) TMZ treatment mice. **Figure S7.** Non-target metabolomics analysis of fecal samples before (Model) and after (TMZ) TMZ treated mice. **(A)** QC samples were clustered very well in PCA score plots derived from LC-MS (+) and LC-MS (-) datasets. (B) Permutation test result (500 times) of OPLS-DA models constructed from LC-MS (+) and LC-MS (-) data. **Figure S8.** Taxonomic distributions of bacteria in TMZ sensitive and non-sensitive mice at phylum level (A) and genus level (B). **Figure S9.** Non-target metabolomics analysis of fecal samples in TMZ sensitive and non-sensitive mice. **(A)** QC samples were clustered very well in PCA score plots derived from LC-MS (+) and LC-MS (-) datasets. (B) Permutation test result (500 times) of OPLS-DA models constructed from LC-MS (+) and LC-MS (-) data. (C) Differently abundant of KEGG pathways of the gut microbiota metabolites in S and NS mice. **Figure S10.** Heatmap of Spearman correlation coefficient between the changed fecal metabolites and bacterial genera. The intensity of the colors represents the degree of association between the level of fecal metabolites and bacterial genera abundance measured by Spearman’s correlations. The *p*-values<0.05 were considered statistically significant, *p<0.05, **p<0.01. **Figure S11.** Relative abundance of bacterial genera/metabolites that was significantly associated with the tumor inhibition rate in TMZ sensitive and non-sensitive mice measured by the Spearman’s correlations analysis. **Figure S12.** Relative expression of key molecules in tryptophan metabolism in the brain and tumor tissue. (A) The relative mRNA expression of AHR and IDO1 in the brain&tumor tissue of Control *versus *Model mice, Sensitive *versus *Non-Sensitive mice. (B) Immunohistochemistry of AHR in Control *versus *Model mice, Sensitive *versus *Non-Sensitive mice. **Table S1.** Differential metabolites in fecal samples between Control (C) and Model (M) group. Only features with VIP > 1, *p *< 0.05 and fold change > 2 were listed. **Table S2.** Differential metabolites in fecal samples before (Model, M) and after TMZ (TMZ, T) TMZ treatment. Only features with VIP > 1, *p *< 0.05 and fold change > 2 were listed.

## Data Availability

The dataset supporting the results of this article was deposited in the Sequence Read Archive (SRA) under BioProject accession code PRJNA885584.
