# Supplementary material for: Gut microbiota mediated the individualized efficacy of Temozolomide via immunomodulation in glioma
Source: J Transl Med. 2023 Mar 16;21:198. doi: 10.1186/s12967-023-04042-5 (PMC10018922; doi:10.1186/s12967-023-04042-5)
Supplement: Supplementary file 1 — Additional file 1: Figure S1. 3D images for glioma orthotopic xenograft mice. Figure S2. The α-diversity indexes of Chao1 (A), Shannon (B) and Simpson (C) of gut microbiota between Control and Model groups. Figure S3. Similarity percentage (SIMPER) analysis based on Bray-Curtis was used to study bacterial species (Top 10) contributing to the variability between Control (C) and Model (M) groups. Figure S4. Non-target metabolomics analysis of fecal samples from Control and Model mice. (A) QC samples were clustered very well in PCA score plots derived from LC-MS (+) and LC-MS (-) datasets. (B) Permutation test result (500 times) of OPLS-DA models constructed from LC-MS (+) and LC-MS (-) data. (C) Differently enrichment of KEGG pathways in Control and Model mice. Figure S5. Taxonomic distributions of bacteria before (Model) and after TMZ (TMZ) treatment at phylum level (A) and genus level (B); (C) Significantly changed bacterial genera between Model and TMZ mice evaluated by MetaStat analysis. Figure S6. Differently abundant of KEGG pathways of the gut microbiota before (Model) and after (TMZ) TMZ treatment mice. Figure S7. Non-target metabolomics analysis of fecal samples before (Model) and after (TMZ) TMZ treated mice. (A) QC samples were clustered very well in PCA score plots derived from LC-MS (+) and LC-MS (-) datasets. (B) Permutation test result (500 times) of OPLS-DA models constructed from LC-MS (+) and LC-MS (-) data. Figure S8. Taxonomic distributions of bacteria in TMZ sensitive and non-sensitive mice at phylum level (A) and genus level (B). Figure S9. Non-target metabolomics analysis of fecal samples in TMZ sensitive and non-sensitive mice. (A) QC samples were clustered very well in PCA score plots derived from LC-MS (+) and LC-MS (-) datasets. (B) Permutation test result (500 times) of OPLS-DA models constructed from LC-MS (+) and LC-MS (-) data. (C) Differently abundant of KEGG pathways of the gut microbiota metabolites in S and NS mice. Figure S10. Heatm [file 12967_2023_4042_MOESM1_ESM.pdf]

Supplementary Information for

Gut microbiota mediated the individualized efficacy of Temozolomide via immunomodulation in glioma

1. Figures

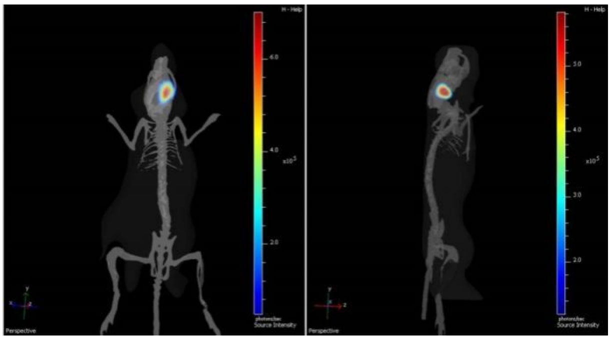

Figure S1. 3D images for glioma orthotopic xenograft mice.

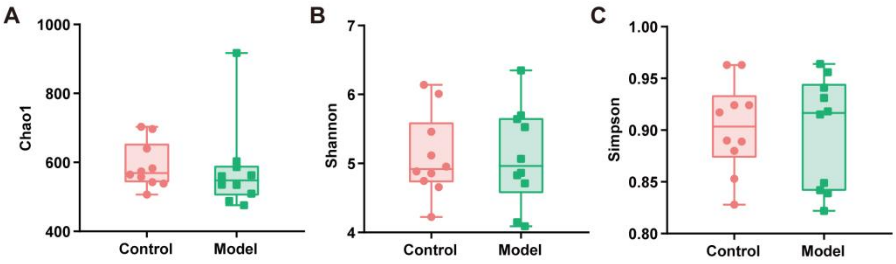

Figure S2. The α-diversity indexes of Chao1 (A), Shannon (B) and Simpson (C) of gut microbiota between Control and Model groups.

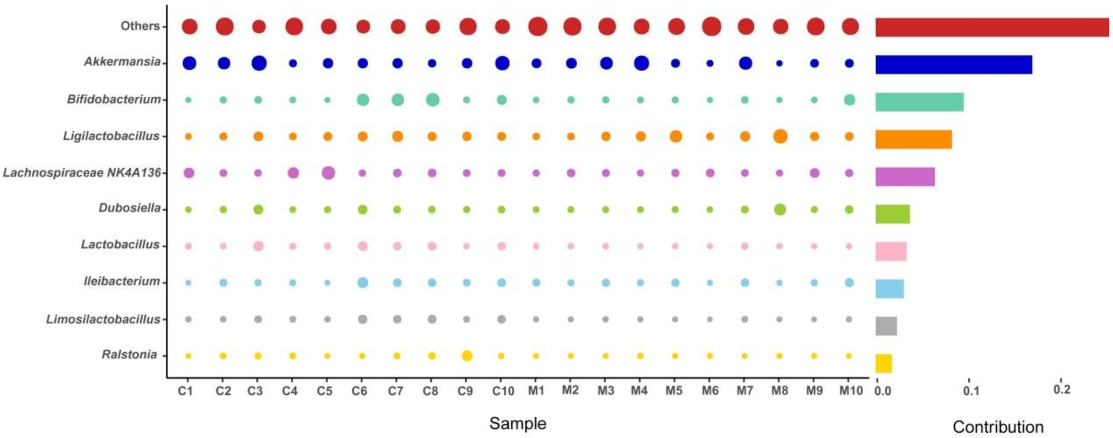

Figure S3. Similarity percentage (SIMPER) analysis based on Bray-Curtis was used to study bacterial species (Top 10) contributing to the variability between Control (C)

and Model (M) groups.

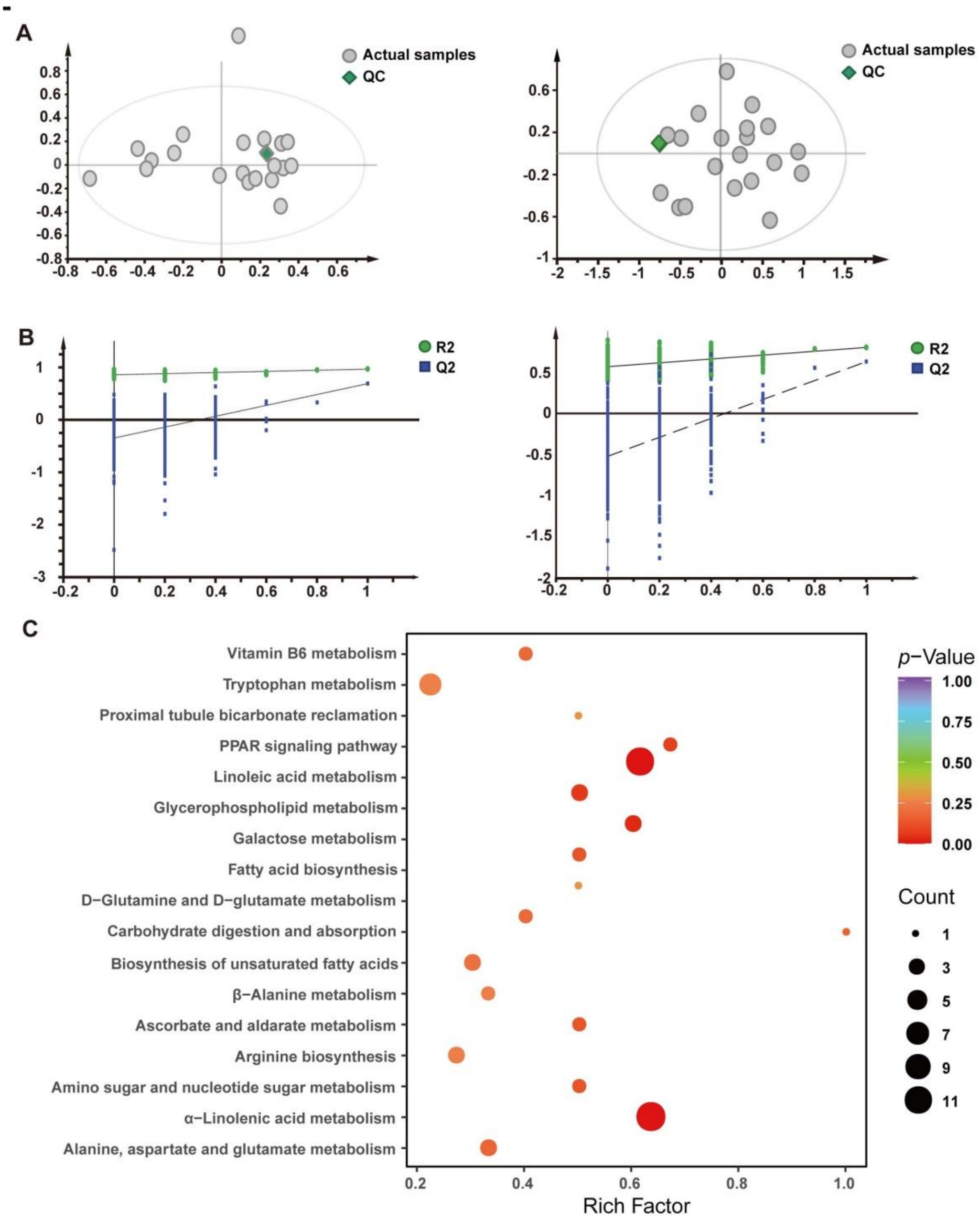

Figure S4. Non-target metabolomics analysis of fecal samples from Control and Model mice. **(A)** QC samples were clustered very well in PCA score plots derived from LC-MS (+) and LC-MS (-) datasets. (B) Permutation test result (500 times) of OPLS-DA models constructed from LC-MS (+) and LC-MS (-) data. (C) Differently enrichment of KEGG pathways in Control and Model mice.

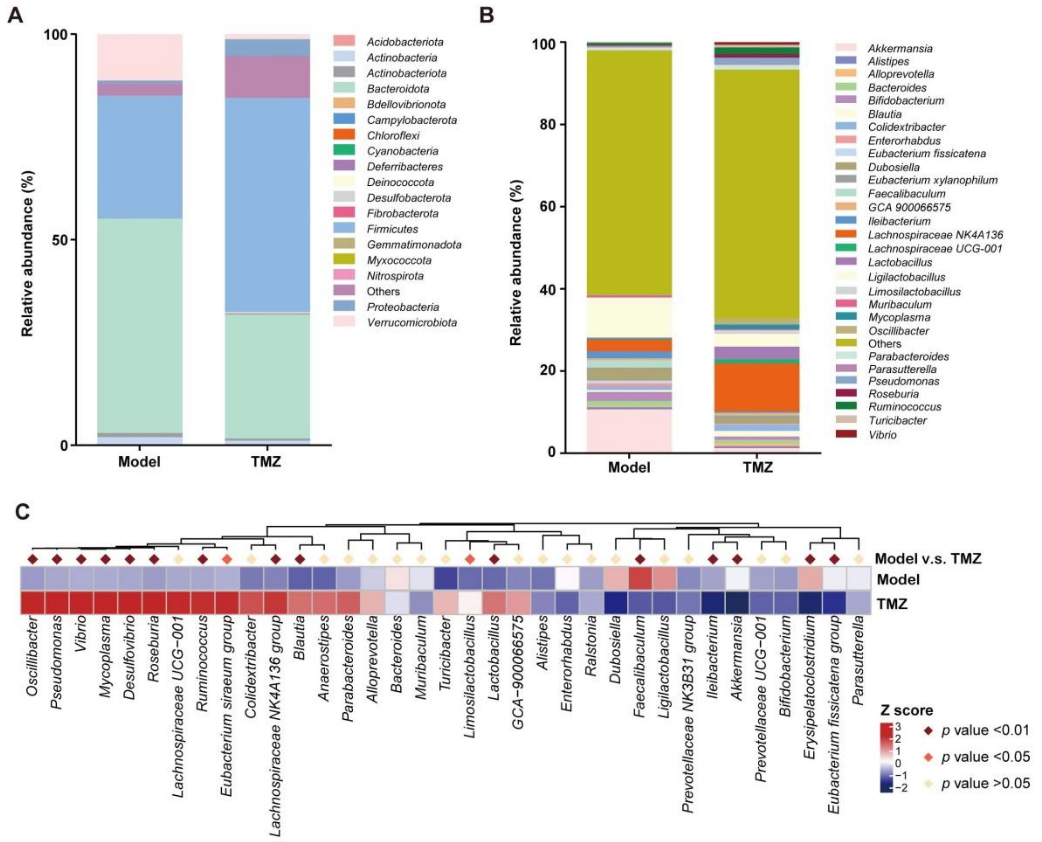

Figure S5 Taxonomic distributions of bacteria before (Model) and after TMZ (TMZ) treatment at phylum level (A) and genus level (B); (C) Significantly changed bacterial genera between Model and TMZ mice evaluated by MetaStat analysis.

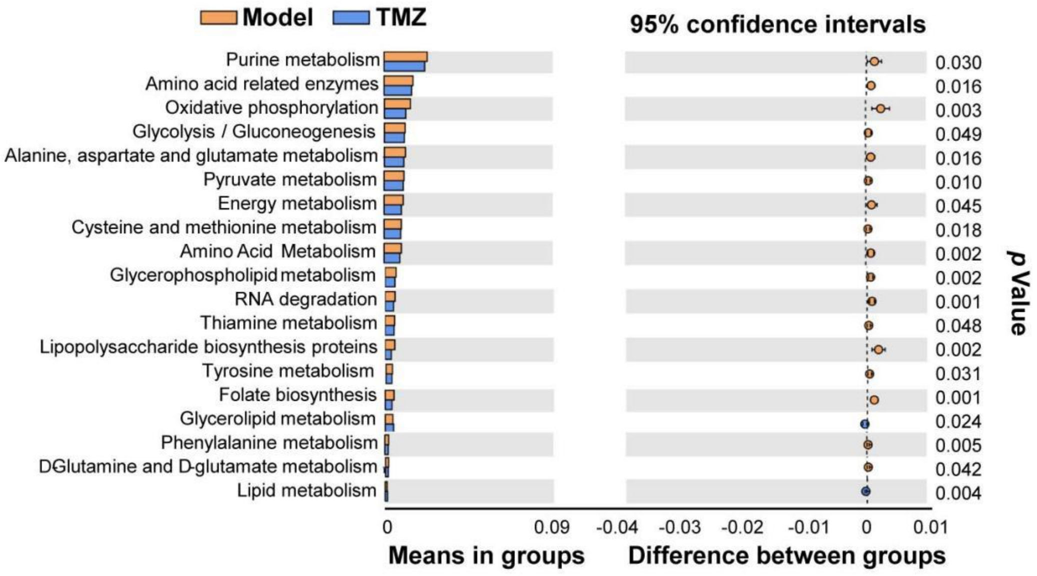

Figure S6 Differently abundant of KEGG pathways of the gut microbiota before (Model) and after (TMZ) TMZ treatment mice.

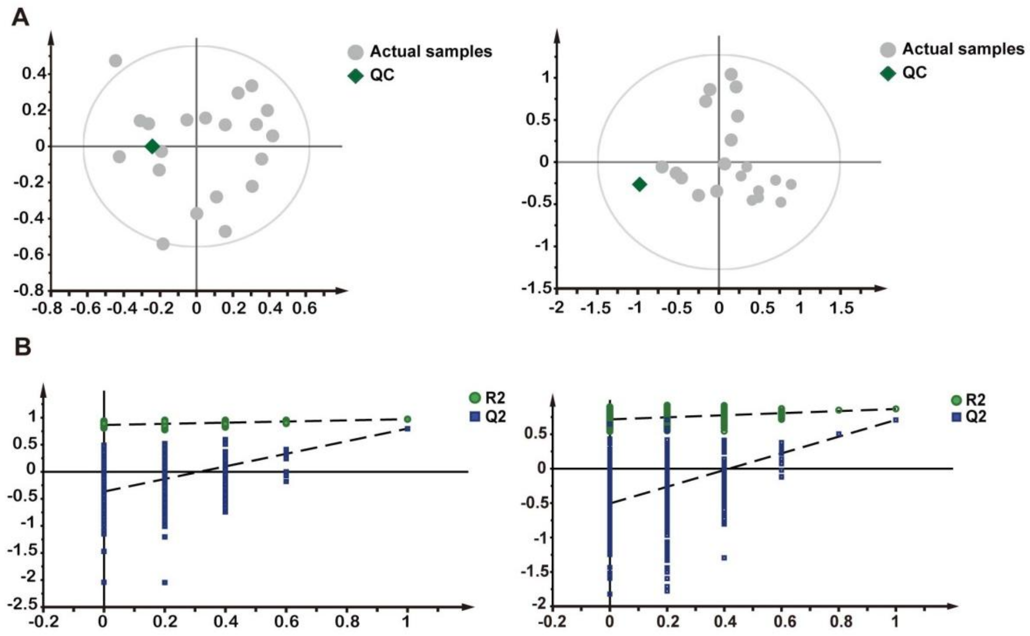

Figure S7 Non-target metabolomics analysis of fecal samples before (Model) and after (TMZ) TMZ treated mice. **(A)** QC samples were clustered very well in PCA score plots derived from LC-MS (+) and LC-MS (-) datasets. (B) Permutation test result (500 times) of OPLS-DA models constructed from LC-MS (+) and LC-MS (-) data.

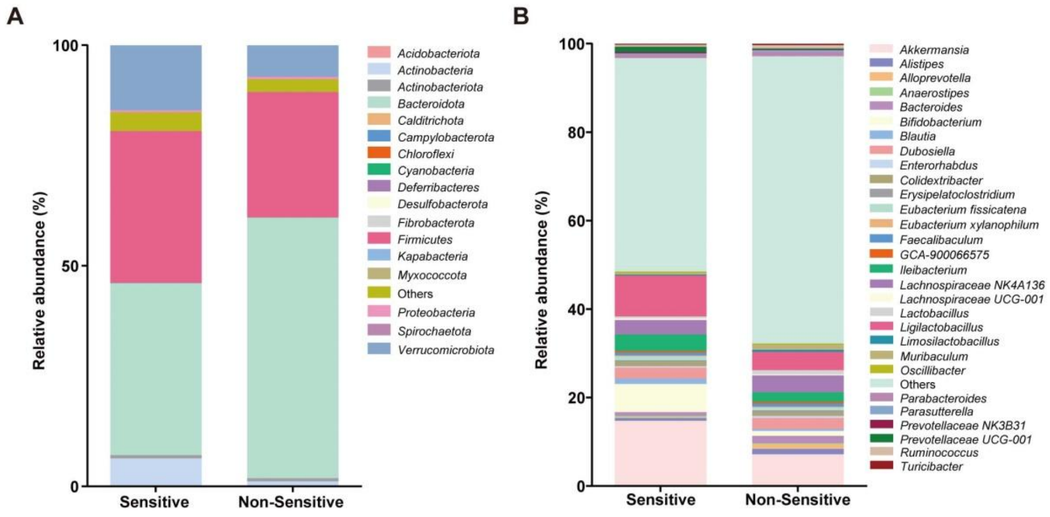

Figure S8 Taxonomic distributions of bacteria in TMZ sensitive and non-sensitive mice at phylum level (A) and genus level (B).

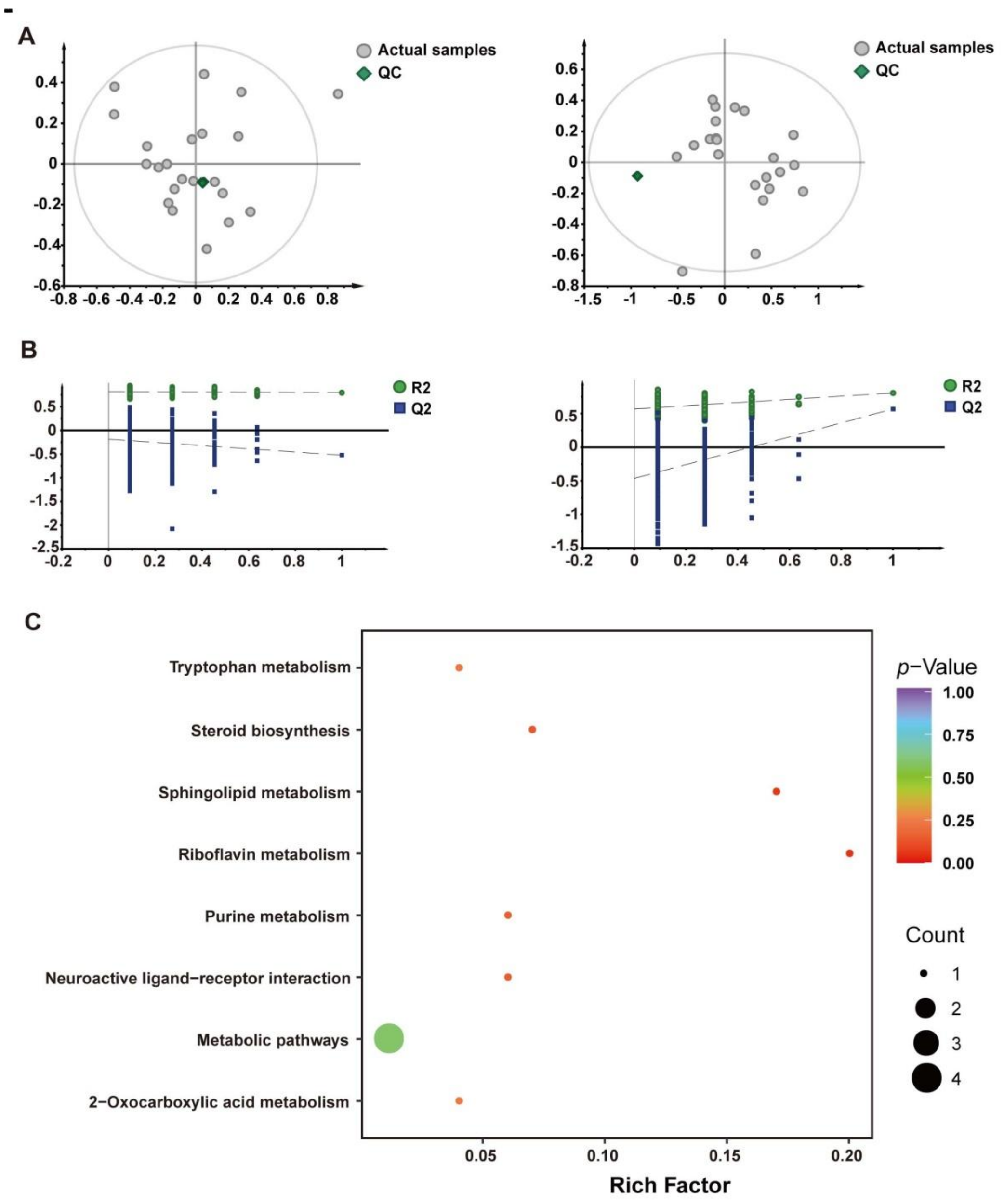

Figure S9 Non-target metabolomics analysis of fecal samples in TMZ sensitive and non-sensitive mice. **(A)** QC samples were clustered very well in PCA score plots derived from LC-MS (+) and LC-MS (-) datasets. (B) Permutation test result (500 times) of OPLS-DA models constructed from LC-MS (+) and LC-MS (-) data. (C) Differently abundant of KEGG pathways of the gut microbiota metabolites in S and NS mice.

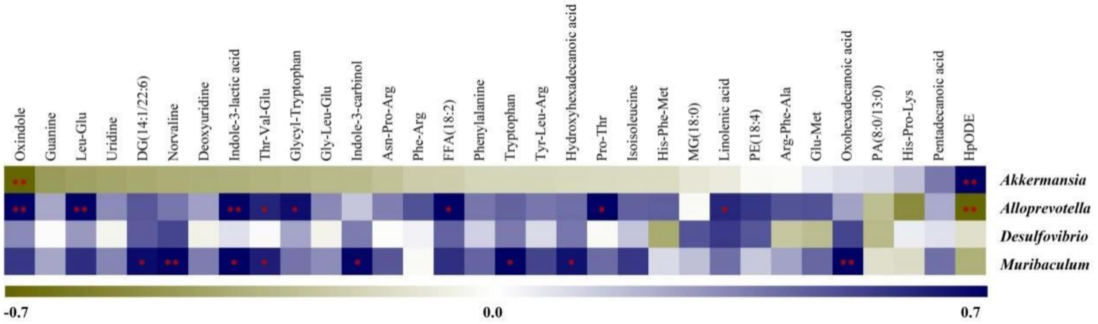

Figure S10 Heatmap of Spearman correlation coefficient between the changed fecal metabolites and bacterial genera. The intensity of the colors represents the degree of association between the level of fecal metabolites and bacterial genera abundance measured by Spearman's correlations. The *p*-values<0.05 were considered statistically significant, *$p<0.05$, **$p<0.01$.

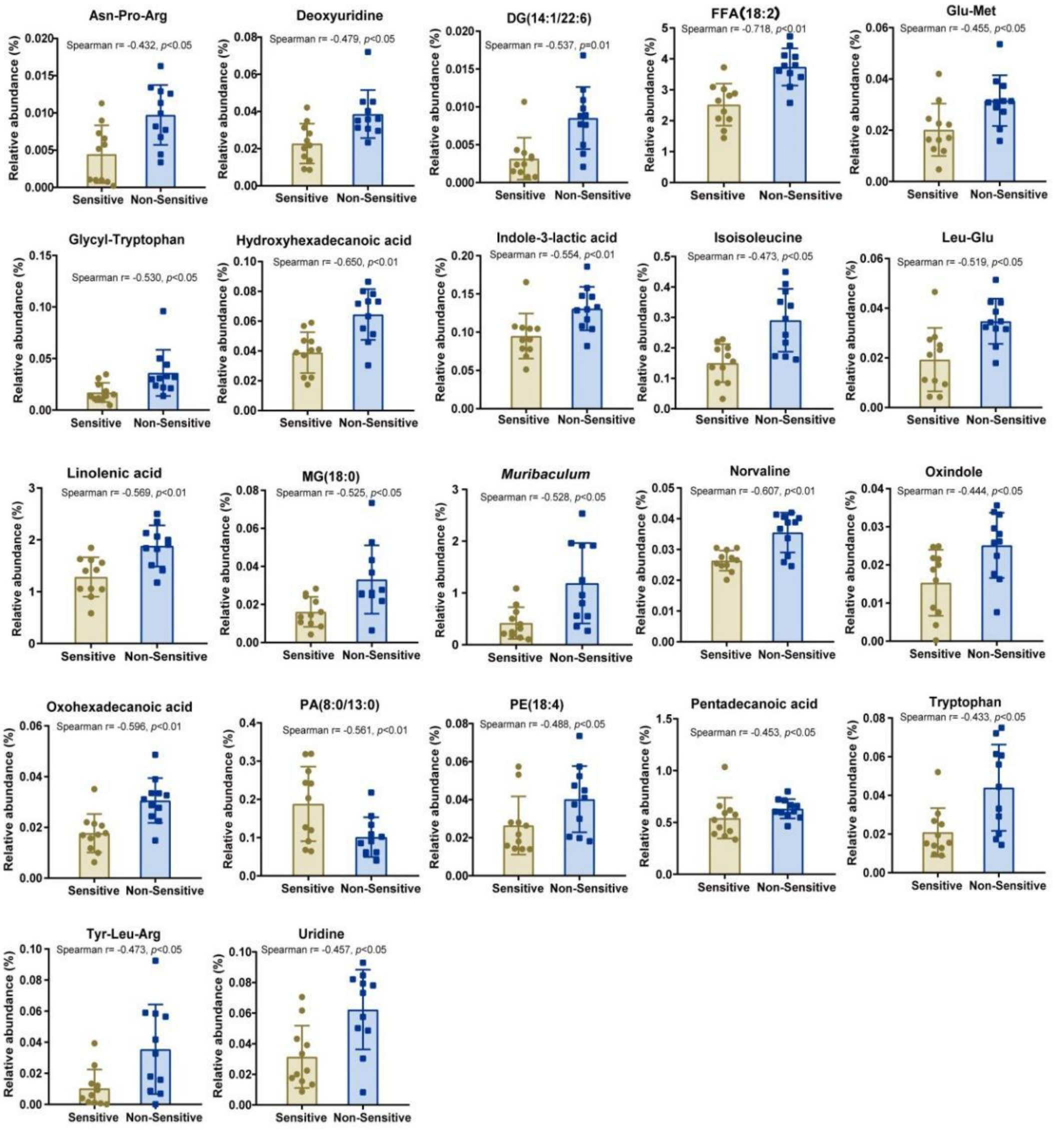

Figure S11 Relative abundance of bacterial genera/metabolites that was significantly associated with the tumor inhibition rate in TMZ sensitive and non-sensitive mice measured by the Spearman's correlations analysis.

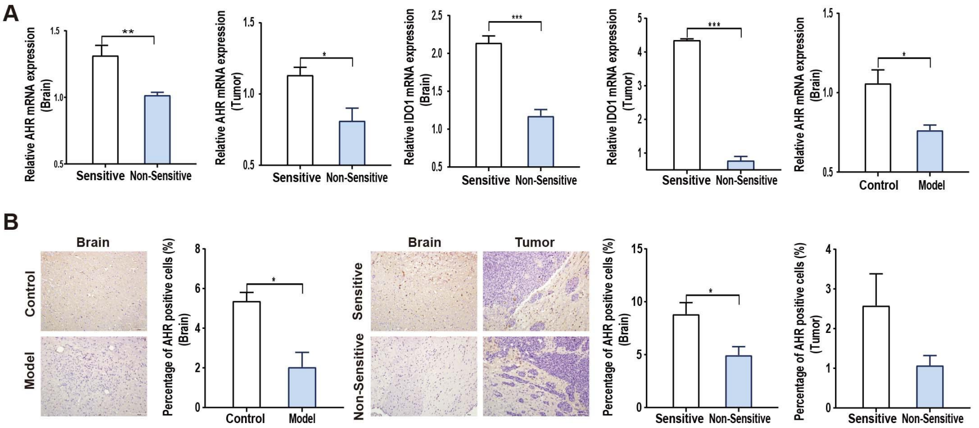

Figure S12 Relative expression of key molecules in tryptophan metabolism in the brain and tumor tissue. (A) The relative mRNA expression of AHR and IDO1 in the brain&tumor tissue of Control *versus* Model mice, Sensitive *versus* Non-Sensitive mice. (B) Immunohistochemistry of AHR in Control *versus* Model mice, Sensitive *versus* Non-Sensitive mice.

2. Tables

Table S1. Differential metabolites in fecal samples between Control (C) and Model (M) group. Only features with VIP > 1, $p < 0.05$ and fold change > 2 were listed.

No.	Metabolites	VIP	*p* value	C/M
1	Dihydroxyhexadecanoic acid	1.15	<0.001	3.29
2	Dihydroxystearate	8.91	<0.001	7.05
3	EpOME	1.74	<0.001	4.98
4	HODE	1.31	<0.001	5.36
5	HpODE	4.00	<0.001	4.27
6	Hydroxycaprylic acid	1.01	<0.001	8.29
7	Methylhexanoic acid	1.41	<0.001	8.73
8	Methylindole	1.55	<0.001	7.06
9	Met-Tyr	1.50	<0.001	6.78
10	Octadecatrienoic acid	1.94	<0.001	4.49
11	Oxo-Dodecenoic Acid	3.13	<0.001	4.51
12	Oxononanoic acid	2.48	<0.001	6.42

13	OxoODE	5.49	<0.001	3.27
14	PA(21:0/14:0)	1.19	<0.001	3.10
15	Sphinganine 1-phosphate	3.01	<0.001	13.22
16	TriHOME	2.68	<0.001	7.01
17	Choline	3.34	0.001	2.89
18	DiHOME	3.11	0.001	3.04
19	Dimethylpentanoic acid	1.06	0.001	3.83
20	FFA(18:0)	9.59	0.001	4.84
21	Glutamic acid	1.86	0.001	0.43
22	Guanidinobutyric acid	1.40	0.001	5.38
23	Heptadecanoic acid	1.45	0.001	4.58
24	HOTrE	6.70	0.001	2.86
25	HpOTrE	3.07	0.001	6.32
26	Hydroxybutyrylcarnitine	1.15	0.001	0.47
27	Leu-Pro	1.50	0.001	2.19
28	Lys-Gln	1.01	0.001	2.62
29	Lysine	1.31	0.001	2.74
30	OxoETE	1.04	0.001	2.24
31	Oxohexadecanoic acid	1.96	0.001	2.99
32	TG(14:0)	2.47	0.001	3.44
33	Cys-Pro	12.03	0.002	2.39
34	Glutathione	1.20	0.002	5.38
35	LPE(15:0/0:0)	5.37	0.002	0.34
36	Octadecadienoic acid	1.94	0.002	2.71
37	Pro-Lys	1.07	0.002	0.31
38	Tyr-Thr	1.23	0.002	3.07
39	Acetyl-hydroxybutanoic acid	1.32	0.003	0.36
40	Aspartic acid	1.33	0.004	2.20
41	HETE	1.29	0.004	2.08
42	Phe-Asn-Glu	1.23	0.004	0.28
43	Tyrosyl-Arginine	1.30	0.004	6.82
44	Hydroxydodecanoic acid	1.58	0.005	0.47
45	Octadecanedioic acid	1.71	0.005	3.24
46	PA(14:1/20:4)	1.13	0.005	6.54
47	Gly-Leu	1.08	0.007	2.30
48	Hyodeoxycholic acid	2.26	0.007	0.41
49	Methylhexadecanoic acid	1.45	0.007	2.66
50	N-Acetyl-aspartic acid	3.15	0.007	2.27
51	Phe-Lys	1.03	0.007	0.30
52	Tryptophan	4.33	0.007	2.38
53	Eicosatrienoic Acid	1.30	0.008	2.98
54	Linoleic acid	2.41	0.008	2.05
55	PA(8:0/13:0)	3.97	0.008	0.45
56	Proline	3.39	0.01	2.49

57	LysoPE(16:0/0:0)	1.86	0.013	0.48
58	Valproic acid	2.042	0.013	3.43
59	PE(16:1/24:0)	1.28	0.016	2.91
60	Ala-gln	3.00	0.023	2.13
61	Threoninyl-Phenylalanine	1.24	0.023	2.02
62	LysoPC(0:0/16:0)	1.05	0.028	2.43
63	DG(18:0/14:1)	1.13	0.041	3.20
64	Leu-Tyr	1.51	0.049	3.13

Table S2. Differential metabolites in fecal samples before (Model, M) and after TMZ (TMZ, T) TMZ treatment. Only features with VIP > 1, $p < 0.05$ and fold change > 2 were listed.

**No.**	Metabolites	VIP	*p* value	**C/M**
1	Acetyl-Aspartic acid	4.48	<0.001	0.34
2	Acetyl-Glutamic Acid	1.31	<0.001	3.16
3	Acetyl-Phenylalanine	1.40	<0.001	0.11
4	Allothreonine	2.54	<0.001	4.73
5	Aspartic acid	1.40	<0.001	0.45
6	Hydroxybutyrylcarnitine	1.30	<0.001	3.38
7	Ile-Met	1.98	<0.001	2.76
8	L-Histidine	1.08	<0.001	8.67
9	LPI(14:0)	1.21	<0.001	12.84
10	Met-Gly-Met	1.03	<0.001	8.63
11	PC(16:0)	10.04	<0.001	5.79
12	Phe-Asp-Phe	1.41	<0.001	2.65
13	Phe-Thr	1.96	<0.001	2.96
14	Pro-Glu-Tyr	1.05	<0.001	2.11
15	Pro-Met-Ser	1.01	<0.001	7.11
16	Valproic acid	1.56	<0.001	5.39
17	Acetyl-Leucine	1.78	0.001	5.33
18	Ala-gln	3.05	0.001	4.82
19	Arginine	1.01	0.001	0.36
20	Dihydroxyindole-2-carboxylic acid	1.29	0.001	3.01

21	FFA(18:0)	9.91	0.001	0.25
22	Glutaric acid	1.51	0.001	6.31
23	Gly-Lys-Ala	2.60	0.001	0.30
24	Hydroxyoctadecanoic acid	9.20	0.001	0.26
25	Indole-2-carboxylic Acid	1.85	0.001	7.36
26	Lys-Lys-Glu	1.17	0.001	8.84
27	LysoPC(20:3)	1.35	0.001	0.41
28	Lys-Val	1.06	0.001	3.58
29	Ornithine	1.18	0.001	2.39
30	Tauroursocholic acid	1.22	0.001	3.59
31	Tyr-Pro-Phe	1.51	0.001	3.19
32	Val-Ser-Gln	1.61	0.001	0.25
33	Leu-Leu	2.17	0.002	2.33
34	Phe-Lys	1.06	0.002	4.88
35	Pro-Thr	1.08	0.002	3.21
36	Thr-Ile-Phe	1.22	0.002	2.73
37	Asp-Arg-Val	1.18	0.003	5.59
38	Cystine	1.64	0.003	7.68
39	LysoPE(15:0)	1.28	0.003	0.23
40	Phe-Asn-Glu	1.43	0.003	7.00
41	Pro-Lys	1.05	0.003	3.41
42	Ile-Leu-Tyr	1.02	0.004	18.29
43	Isobutyric acid	1.14	0.004	2.84
44	Oxohexadecanoic acid	1.14	0.004	0.48
45	Gln-Arg-Ile	1.22	0.005	2.59
46	Lys-Asp-Gly	1.12	0.005	4.08
47	Vulpinic acid	1.15	0.005	3.75
48	Carnitine C9:1	1.29	0.007	4.11
49	Lys-Pro-Arg	1.17	0.007	2.30
50	Gln-Met-Lys	1.29	0.008	2.10
51	LPC(22:6)	1.68	0.008	2.21
52	Pro-Thr	1.38	0.008	2.28
53	Hydroxy-2-methylbutyric acid	1.39	0.01	4.33
54	Hydroxyhexadecanoic acid	3.45	0.01	0.24
55	LPE(16:0)	5.42	0.01	2.33
56	Indole-4-carboxaldehyde	1.73	0.013	2.16
57	Isohyodeoxycholic acid	3.23	0.013	0.25
58	Ketodeoxycholic acid	2.19	0.013	2.43
59	PE(22:6)	1.04	0.016	0.40
60	Fluoroindole-2-carboxylic acid	1.93	0.019	2.74
61	Guanine	4.03	0.028	0.34

Table S3. Differential metabolites in fecal samples between TMZ sensitive (S) and non-sensitive (NS) mice. Features with VIP > 1 and $p < 0.05$ were listed.

**No.**	Metabolites	VIP	*p* value	**C/M**
1	FFA(18:2)	11.51	0.001	0.67
2	DG(14:1/22:6)	1.37	0.003	0.37
3	Hydroxyhexadecanoic acid	1.47	0.003	0.60
4	Oxohexadecanoic acid	1.12	0.003	0.58
5	Isoisoleucine	3.39	0.004	0.52
6	Linolenic acid	7.96	0.004	0.68
7	Deoxyuridine	1.10	0.005	0.59
8	Leu-Glu	1.28	0.005	0.56
9	Norvaline	1.66	0.005	0.74
10	Asn-Pro-Arg	1.40	0.008	0.46
11	Glycyl-Tryptophan	1.02	0.008	0.47
12	Indole-3-lactic acid	2.09	0.008	0.73
13	MG(18:0)	2.69	0.008	0.49
14	Tryptophan	1.27	0.008	0.48
15	Oxindole	1.42	0.011	0.61
16	Uridine	1.44	0.011	0.50
17	Phenylalanine	2.42	0.017	0.57
18	Tyr-Leu-Arg	1.44	0.02	0.29
19	Arg-Phe-Ala	1.36	0.023	0.40
20	Guanine	3.57	0.023	0.49
21	Glu-Met	1.17	0.028	0.64
22	HpODE	1.62	0.028	1.87

23	PA(8:0/13:0)	4.43	0.028	1.85
24	Pro-Thr	1.15	0.028	0.60
25	Gly-Leu-Glu	1.46	0.033	0.62
26	His-Pro-Lys	1.26	0.033	2.53
27	Indole-3-carbinol	1.12	0.039	0.69
28	Phe-Arg	1.27	0.039	0.50
29	His-Phe-Met	2.40	0.045	0.69
30	PE(18:4)	1.09	0.045	0.66
31	Pentadecanoic acid	2.98	0.045	0.86
32	Thr-Val-Glu	1.44	0.045	0.73
